# What Is the Diagnosis in a Young Woman With Painful Proximal Nail Fold Inflammation of Both Great Toes?

**DOI:** 10.1002/ccr3.72617

**Published:** 2026-04-24

**Authors:** Aya Awwad, Ali Hamieh, Ali Fakih

**Affiliations:** ^1^ Dermatology, Faculty of Medical Sciences Lebanese University Beirut Lebanon; ^2^ Dermatology, Baabda University Hospital Baabda Lebanon; ^3^ Dermatology, Hammoud Hospital University Medical Center Saida Lebanon

**Keywords:** ingrown nail, nail disorders, nail pain, nail plate avulsion, proximal nail fold inflammation, retronychia

## Abstract

Retronychia should be suspected in patients presenting with painful proximal nail fold inflammation of the great toe, particularly when nail growth has slowed or ceased. Early recognition prevents misdiagnosis as paronychia or onychomycosis. Complete nail plate avulsion remains the most effective treatment for symptom resolution.

## 1 Clinical Question

A 32‐year‐old woman presented with a 2‐month history of periungual pain affecting both great toes, associated with progressive nail discoloration and lack of response to oral antifungal and antibiotic therapies. What is the most likely diagnosis?

**Answer:** Retronychia.

## 2 Case Description and Teaching Point

**FIGURE 1:**
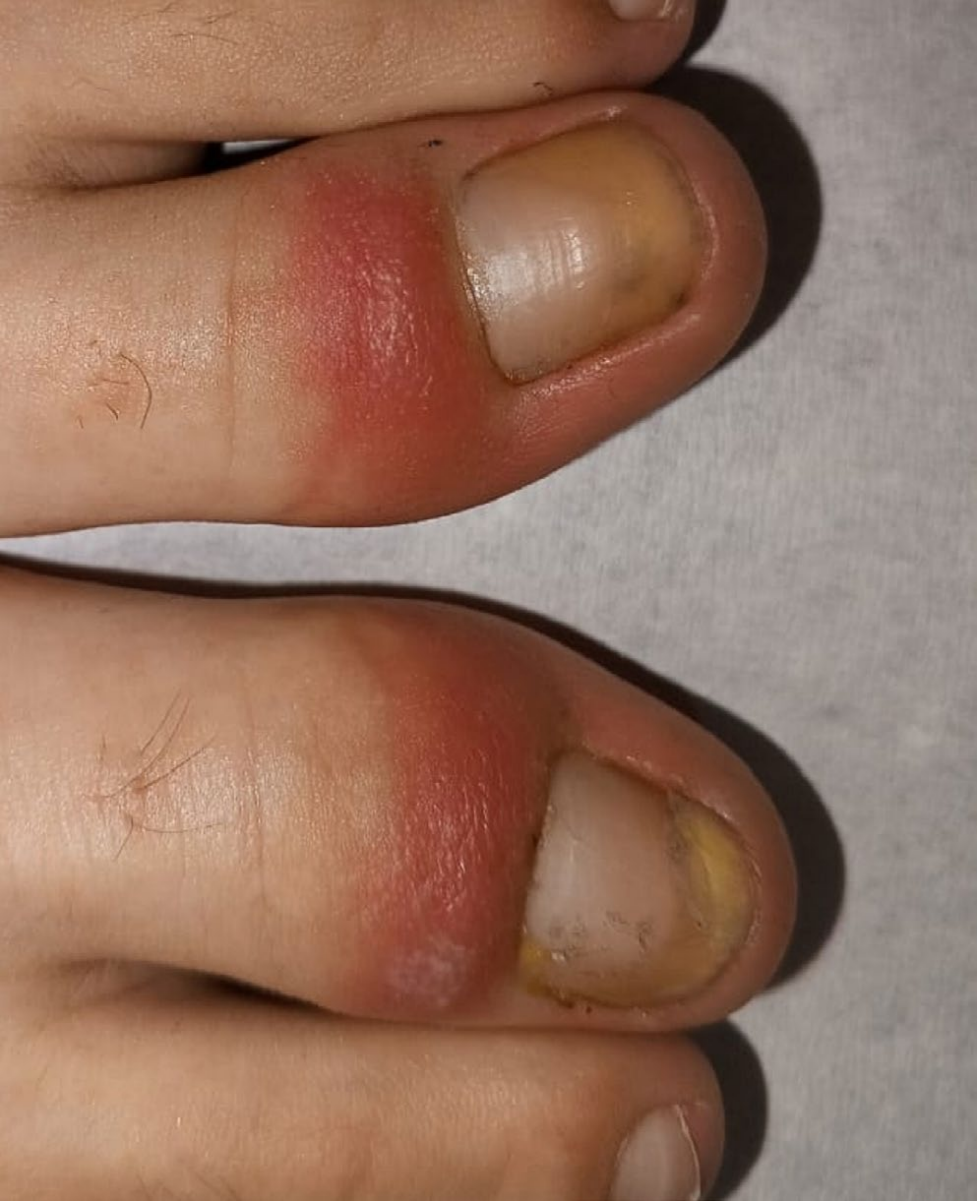
Close‐up view showing erythema and edema of the proximal nail fold with loss of the cuticle and a thickened, yellowish, partially detached nail plate.

Clinical examination revealed marked erythema and edema of the proximal nail folds, loss of the cuticle, and yellowish, thickened, partially detached nail plates with subungual exudate but no granulation tissue, involving both great toenails (Figure 1). The remaining toenails and all fingernails were clinically normal, and cutaneous examination was unremarkable. The medical history, characteristic clinical findings, absence of mycological evidence, and lack of response to oral terbinafine and antibiotic therapy supported the diagnosis of retronychia, a proximal ingrown nail disorder.

Retronychia is an uncommon proximal ingrown nail disorder that predominantly affects young women and is frequently triggered by repetitive microtrauma, leading to temporary interruption of nail matrix activity. Incomplete shedding of the old nail plate results in its proximal embedding within the nail fold, preventing normal forward growth of the new nail plate and causing persistent periungual inflammation [1, 2].

Clinically, retronychia should be suspected in patients presenting with painful proximal nail fold erythema and edema, loss of the cuticle, yellowish thickened nail plate, and notably, slowing or cessation of nail growth. Although ultrasonography can support the diagnosis by demonstrating multiple overlapping nail plates, the diagnosis remains primarily clinical in typical presentations.

Retronychia is often misdiagnosed as onychomycosis or acute paronychia, leading to ineffective antifungal or antibiotic therapy. Unlike onychomycosis, retronychia characteristically presents with proximal nail fold inflammation and pain, accompanied by halted nail growth.

Complete nail plate avulsion, performed by dermatologists, remains the most effective treatment and allows symptom resolution and normal regrowth of the nail. Early recognition is essential to avoid unnecessary treatments and ensure appropriate management [3].

## Author Contributions

**Aya Awwad:** data curation, investigation, writing – original draft. **Ali Hamieh:** data curation, investigation, writing – original draft. **Ali Fakih:** conceptualization, supervision, validation, writing – review and editing.

## Funding

The authors have nothing to report.

## Consent

Written informed consent from the patient was obtained for publication.

## Conflicts of Interest

The authors declare no conflicts of interest.

## Data Availability

Data sharing not applicable to this article as no datasets were generated or analysed during the current case.
